# Diet of the Insular Lizard, *Podarcis lilfordi* (Günther, 1874): Complementary Morphological and Molecular Approaches

**DOI:** 10.3390/ani13030507

**Published:** 2023-02-01

**Authors:** Iris Alemany, Ana Pérez-Cembranos, José A. Castro, Antònia Picornell, Valentín Pérez-Mellado, Cori Ramon

**Affiliations:** 1 Department of Biology, University of the Balearic Islands, Ctra. Valldemossa km 7′5, 07122 Palma de Mallorca, Spain; 2 Department of Animal Biology, Universidad de Salamanca, 37071 Salamanca, Spain

**Keywords:** Balearic Islands, diet analysis, metabarcoding, morphological identification, trophic ecology, *Podarcis*, Lacertidae

## Abstract

**Simple Summary:**

Traditionally, the diets of lizards and other small vertebrates have been studied using invasive techniques, such as the analysis of gastric contents obtained by dissection or stomach-flushing. Nowadays, the morphological analysis of the remains contained in feces is commonly used. However, these techniques require a great deal of experience to identify prey remains, and some prey items may be left undetected in the analyses. Recently, the use of molecular techniques has made it possible to identify prey from feces, thus significantly expanding the diversity of identified prey. Despite this, the molecular analysis of diets also has limitations, since it does not allow an adequate quantification of the contribution of each prey type, and, on occasion, some prey may not leave traces of DNA detectable in the feces. For this reason, here we propose a study of the diet in which the two methods of fecal analysis are complementarily used: morphological identification and identification by means of DNA analysis.

**Abstract:**

The diets of insular lizards are extremely varied, depending on the different environmental characteristics of each island population. This is particularly evident in the case of the populations of small coastal islets of the Balearic Islands, where the Balearic lizard, *Podarcis lilfordi*, is found. The study of trophic ecology carried out by means of traditional tools, such as morphological analysis of feces, has made it possible to detect numerous prey and nutritional elements. However, these methods are clearly insufficient, as some rare groups are not detected. It is also difficult to identify remains of marine subsidies or of foods contributed to these small islands by other predators, such as seabirds. The current study demonstrates the advantages of combining morphological diet analysis with the molecular study of individual feces samples obtained from the same populations. We obtained a greater diversity of prey groups using the combined methodologies, with each method identifying prey items that were not detected using the other method. Particularly, the study of diets at the molecular level identified plant species consumed by lizards that were, occasionally, not identified in morphological analyses. Conversely, the traditional morphological study of an equivalent number of fecal samples allowed for the identification of several prey groups that had not been detected in the molecular study. From this viewpoint, the advantages and disadvantages of each methodology are discussed.

## 1. Introduction

Lizard diets can be complex, and feeding habits are expected to vary among species depending on evolutionary history, microhabitat characteristics, and prey availability [1]. Most lizard species are generalist predators [2]. Traditionally, they were considered insectivores, although collectively they consume a much wider variety of food types, including plants [3]. Several of the available dietary studies of *Podarcis* species are based on morphological analyses of stomach contents from sacrificed lizards and/or from preserved specimens of herpetological collections [4]. Alternative methods for studying diet include stomach flushing [5,6] and direct observation of feeding [7]. The use of stomach contents obtained by dissection raises ethical problems, as it requires the sacrifice of specimens to be studied and, furthermore, is not desirable in protected species whose populations may be threatened. Similarly, stomach flushing can be considered an invasive and disruptive technique [8], undesirable for the study of protected species. For this reason, many current studies are carried out using feces obtained directly from lizards or from the environment, in some cases adding information obtained from direct observations [9]. This allows a detailed study of the diet, even with the identification of soft-bodied prey, such as insect larvae [10]. The major drawback of studying diet using fecal samples is that it requires a great deal of experience to identify animal prey and plant remains, such as seeds and pollen grains, and is, therefore, a time-consuming process restricted to specialists.

Some common prey of lizards go through different developmental stages (e.g., soft-bodied beetle larvae) and may not be identifiable at lower taxonomic levels; furthermore, digestive processes submit prey tissue to intense deterioration. Consequently, the absence of hard parts can make identification difficult [11]. In the case of vegetal matter, it is often necessary to employ microscopic techniques for staining plant tissues, further complicating the task of taxonomic identification.

Current DNA-based methods of taxonomic identification, such as metabarcoding, are useful for the study of trophic spectra [12]. The combination of different barcodes could contribute to a more effective taxonomic assignment. Prey-specific DNA sequences, recovered from gut or fecal samples, are usually short (100–400 bp approx.) and low-quality owing to DNA degradation during digestion [13,14]. When diet DNA is degraded, mini-barcodes are required [15]. Additionally, degraded prey DNA commonly is mixed with prevalent high-quality DNA from the predator [16]. Different methods to prevent DNA co-amplification exist. Molecular methods remove some limitations of traditional techniques, but present others, especially the requirement of technical expertise, economic cost, and availability of appropriate databases. For this reason, DNA-based analyses should be combined with conventional dietary analyses and ecological research to allow the effective taxonomic identification of prey [17].

In the present study, we applied the traditional morphological prey identification from fecal samples and metabarcoding identification from fresh feces to assess diet partitioning and feeding strategies in five populations of the Balearic lizard *Podarcis lilfordi* (Günther, 1874). The Balearic lizard is one of the two endemic species inhabiting the Balearic Islands. The species is only present in coastal islets around the Mallorca and Menorca islands, as well as in the Cabrera archipelago [18]. Because of its restricted distribution and the situation of several populations, the Balearic lizard is considered an endangered species ([18] and references therein). We selected this species for our study because our previous knowledge of its omnivorous diet, comprising a wide range of prey and vegetal matter [4,9,18,19].

## 2. Materials and Methods

### 2.1. Study Area and Sampling

**Figure 1:**
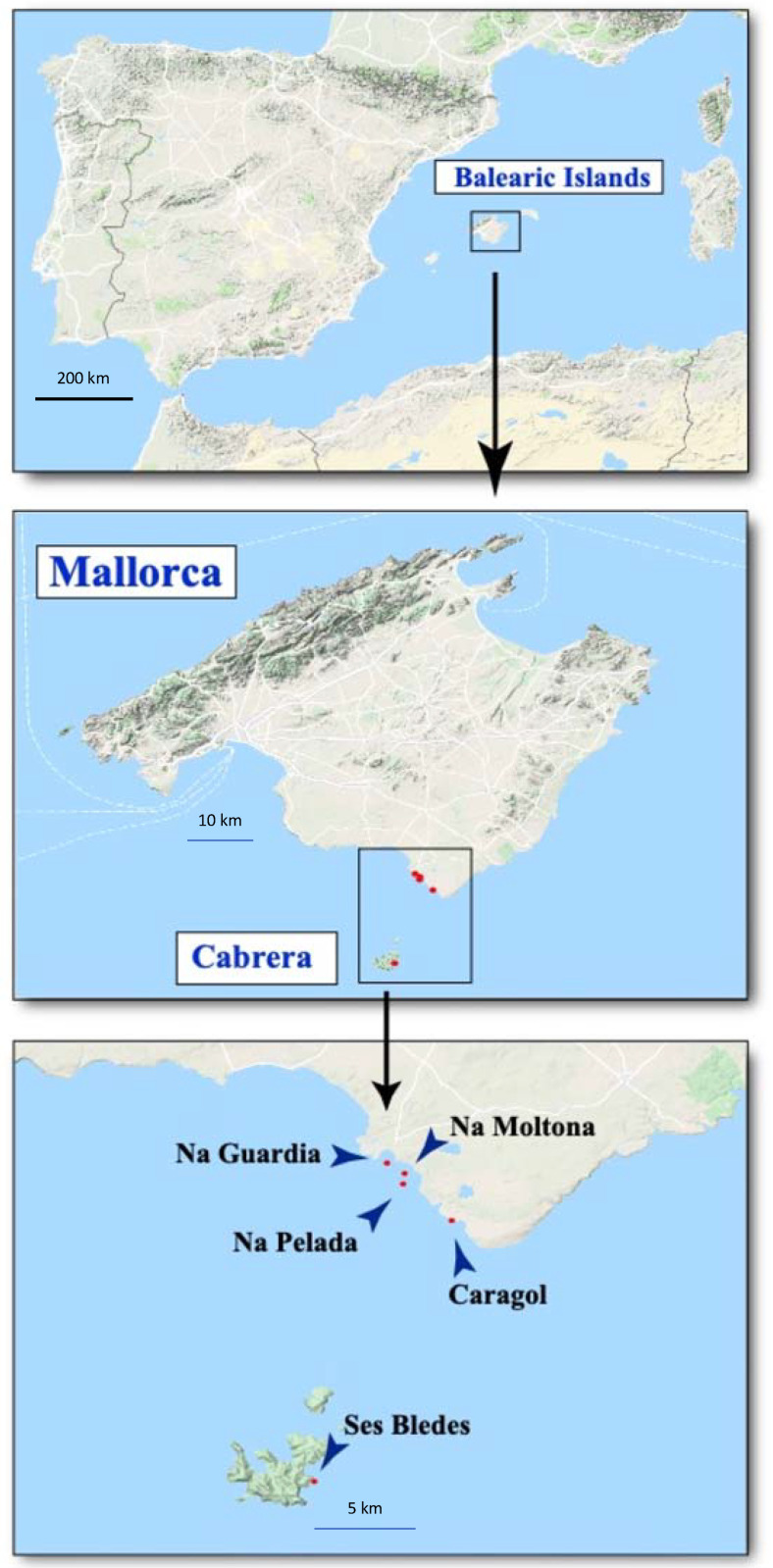
Maps of Mallorca and the Cabrera archipelago showing the location of the sampled *Podarcis lilfordi* (blue arrows). Maps were obtained with Google Maps (Map data 2020 Google) using the function ‘get_map’ in the package ‘ggmap’ version 3.0.0.902 in R version 3.6.3.

For the metabarcoding identification, a total of 37 fresh fecal samples from *P. lilfordi* were collected during the summer of 2018 in the Balearic Islands. From the Cabrera archipelago, at the islet of Ses Bledes, 5 females and 1 male were sampled, and from the islets offshore Mallorca, 31 samples were taken from 15 females and 16 males: Caragol (3/5), Na Guardia (3/4), Na Moltona (5/3), and Na Pelada (4/4) (Figure 1). Lizards were captured by noosing and fresh droppings were directly obtained with a gentle abdominal massage and stored in absolute ethanol vials. Lizards were immediately released at the point of capture. The samples were preserved at 4 °C in the field; upon arrival to the laboratory, they were stored at −20 °C until DNA extraction [12].

Additionally, a total of 138 different individual feces samples, from the same season and localities as the molecular samples, were included in the morphological analysis. For these samples, we collected feces from individual lizards, as well as from the ground.

### 2.2. Molecular Study of the Diet

#### 2.2.1. DNA Extraction and Library Preparation

Total DNA was extracted from individual samples using the Isolate Fecal DNA Kit (Bioline, London, UK) following the manufacturer’s protocol. Samples were submitted to the Roy J. Carver Biotechnology Center (University of Illinois, Ill., USA) for amplification in a microfluidic high-throughput multiplexed PCR platform (Fluidigm). For animal prey detection, we used mitochondrial cytochrome oxidase I (*COI*) for the following primer pairs: mlCOlintF/jgHCO2198 (5′-GGWACWGGWTGAACWGTWTAYCCYCC-3′/5′-TANACYTCNGGRTGNCCRAARAAYCA-3′ [20,21] and specific arthropod ArtF11/ArtR17 (5′-GGNKYNGGNACWGGATGAACWGTNTAYCCNCC-3′/5′-GGRTCAAAAAATGAWGTATTHARATTTCGRTCWGTTA-3′ [22]. To study the ingested plants, the following *rbcL* and *psbA-trnH* chloroplast markers were used: psbA3_f/trnHf_05 (5′-GTTATGCATGAACGTAATGCTC-3′/5′-CGCGCATGGTGGATTCACAATCC-3′; [23,24] and rbcLa_F/rbcLa_R (5′-ATGTCACCACAAACAGAGACTAAAGC-3′/5′-GTAAAATCAAGTCCACCRCG-3′ [25,26]. CS1 and CS2 Fluidigm universal tags and barcode labels specific to each sample and Illumina adapters i5 and i7 were used. The resulting amplicons were validated on a Fragment Analyzer (Agilent) using the HS NGS kit (DNF-474-33). Sequencing was conducted on an Illumina MiSeq v2 platform yielding 2 × 250 paired-end reads. Regarding animal prey, a blocking primer-targeting region within *COI* was used to minimize host amplification. For the design, we used *COI* sequences of the most commonly consumed prey reported from previous studies of Balearic lizards [9] available at Barcode of Life Data System (BOLD; http://www.boldsystems.org, accessed on 1 September 2022) and *COI* fragments of *P. lilfordi* available through the GenBank accession codes in [18].

#### 2.2.2. Sequence Analyses and Taxonomic Assignment

Micca version 1.7.2 [27] was used for merging, trimming, filtering, and OTU picking (see [12] for more details). For the taxonomic assignment (Table 1), the resulting DNA sequences (OTUs) were joined into 98% similarity clusters with Usearch version 10.0.240_i86osx32 [28]. Consensus sequences (centroid) were defined for each cluster and were used to download, from public DNA databases (GenBank and rrnDB), the 1000 most similar sequences.

Individual centroids were analyzed using all the sequences forming the cluster and the 1000 most similar sequences. DNA matrices were aligned with MAFFT [29] and used for phylogenetic inference employing IQTREE [30]. Trees were explored in FigTree [31] to establish the systematic position of the diet sequence to the highest taxonomic rank according to bootstrap support values ≥ 70% [32]. Information from multiple markers targeting the same group of organisms (i.e., animals or plants) was merged following the multi-marker metabarcoding approach described in [33].

Finally, taxonomic assignments and current nomenclature from metabarcoding (Table 1) were checked with available information provided in the taxonomic literature of plants and invertebrates recorded in the Balearic Islands (List A1 (prey references) and List A2 (plant references) from Appendix A).

### 2.3. Morphological Study of the Diet

We analyzed fecal pellets under a binocular dissecting microscope. Diet reconstruction was based on a meticulous pellet analysis that is highly comparable to diet reconstructions based on gastric contents, with soft-bodied prey and, particularly, insect larvae being equally represented in fecal pellets and gut contents [10]. Each scat was spread in a thin layer of less than 0.5 mm over the entire surface of a Petri dish with some drops of 70% ethanol. Plant families were arranged according to Cronquist [34] and Bremer et al. [35]. Prey remains were visually identified up to their order or, in some cases, family level (Table 2). Prey number for each fecal pellet was conservatively estimated by counting only easily identifiable remains. We detected the consumption of plant remains in morphological analysis, but without any taxonomic assignment (see [9] for more details).

### 2.4. Diet Comparison

Because fecal pellet sample sizes were different between morphological and molecular analyses, we tested for the contribution of each prey type to the diet between molecular and morphological samples only, using a subset of 10 feces from larger morphological samples (Table 2). This subset was randomly extracted from each population sample. Because we could not estimate the quantitative contribution of each prey type from molecular samples, we compared only their presence [9] in the feces.

For the five populations, the presence of different prey types found with morphological and molecular methods (Table 2) of analysis were compared with a permutational multivariate analysis of variance (permutational MANOVA, [36]), using the ‘adonis’ function from ‘vegan’ R package [37]. The multivariate homogeneity of group dispersions (variances) was tested with the function ‘betadisper’, a multivariate analogue of Levene’s test for homogeneity of variances.

We estimated and compared diet diversities using the approach proposed by Pallmann et al. [38]. We converted “raw” Simpson or Shannon indices into “true” diversities, which belong to the same mathematical family. Thus, different measures of area were regarded as special cases of Hill’s general definition of diversity measures [39]. Two-tailed tests for integral Hill numbers were performed to compare diets obtained from the different analytical methods. This selection includes the transformed versions of the two following indices: the Shannon entropy index, H_sh_ (q→1), and the Simpson concentration index, H_si_ (q = 2, [40]). We performed 5000 bootstrap replications to obtain reliable *p*-values [41]. Methods described here were implemented in the R package “simboot” [42] and are fully described in [38]; more details of dietary analysis can be found in [9].

The frequency of each prey type present in the fecal samples was compared with the Fisher exact tests. All calculations were performed in R version 4.0.3 [43].

## 3. Results

### 3.1. Morphological and Molecular Diet Compositions

**Figure 2:**
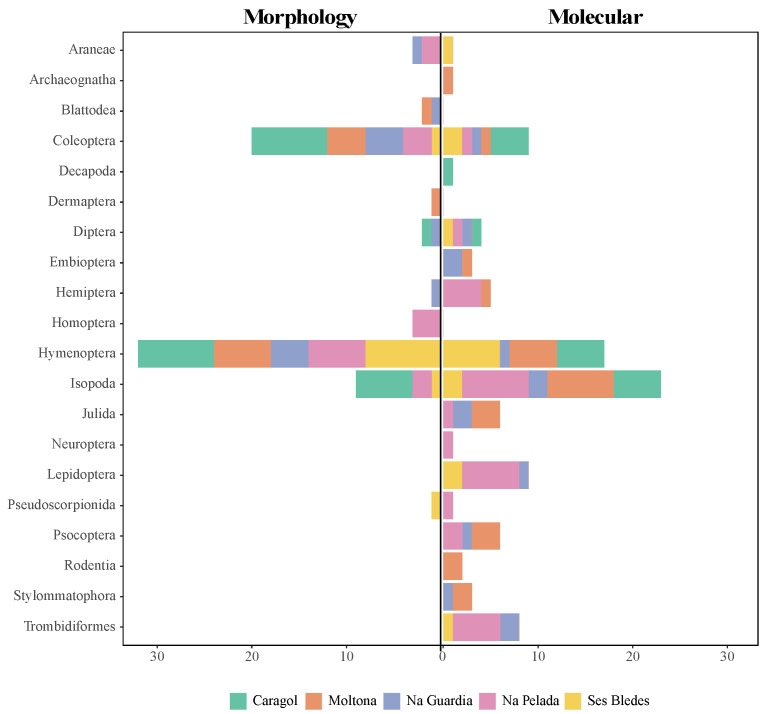
Frequencies of occurrence of each order of animals in the diet composition of *Podarcis lilfordi* through morphological and molecular analysis.

**Figure 3:**
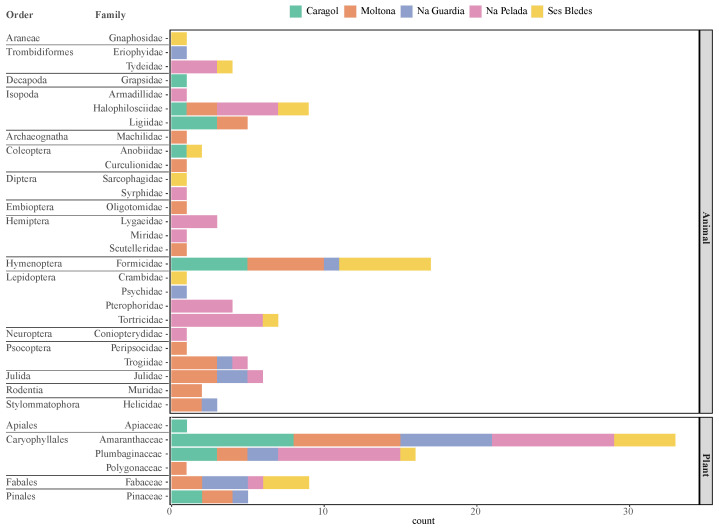
Percentage of occurrence of each family of animals or plants in the diet composition of *Podarcis lilfordi* through molecular analysis. All populations pooled.

**Figure A1:**
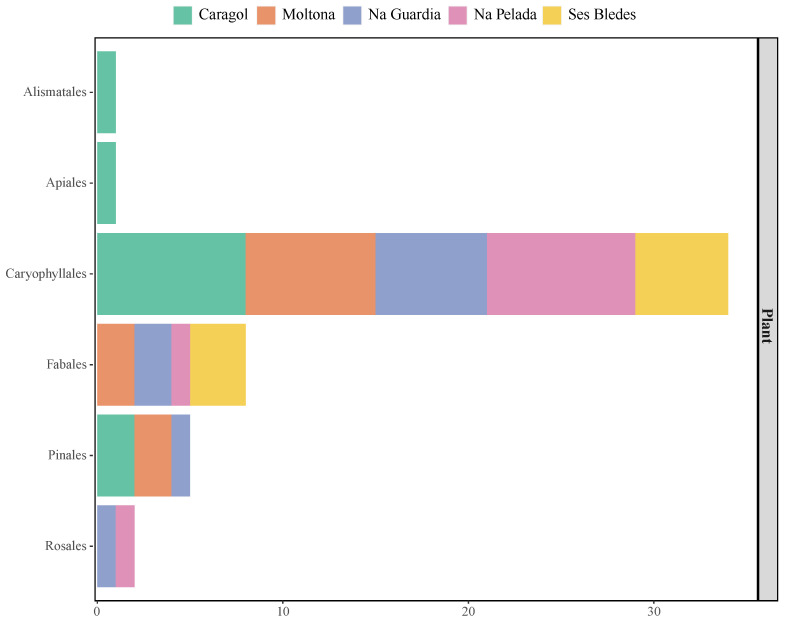
Frequencies of occurrence of each order of plants in the diet composition of *Podarcis lilfordi* through molecular analysis.

In molecular analysis, a total of 57 prey taxa were identified corresponding to 17/5 orders and 27/6 families (Animals/Plants, respectively) (Table 1). The most common orders identified for animal components were Isopoda, Hymenoptera, Lepidoptera, and Coleoptera (Figure 2). Within order Hymenoptera, Formicidae was the only represented family, while Halophilosciidae was the most abundant of the Isopoda orders (Figure 3). In morphological analysis, we identified 9 different prey taxa in the random sample of 10 fecal pellets (Table 2). With regards to plant components, Caryophyllales (Figure A1) was the most abundant Order, represented by Amaranthaceae and Plumbaginaceae families (Figure 3).

### 3.2. Comparison of Morphological and Molecular Analytical Methods

Comparisons were made, taking into account only ten randomly-selected morphological molecular and molecular samples. Results are represented in Figure 2.

We found overall significant differences in diets from morphological and molecular analyses (adonis, F_1,79_ = 6.7728, *p* = 0.0009, with homogeneous variances, F_1,79_ = 0.022, *p* = 0.88). However, we obtained variable results from the comparison of subsamples from each population. At the Ses Bledes islet, we did not find significant differences in the presence of different taxa between the two samples of ten fecal pellets (Fisher Exact Test, *p* = 0.3038), because the diet comprised mainly ants (Table 1 and Table 2). Regardless, the detection of different prey items, using the two methodologies, was extremely different, with four groups of prey not detected by molecular analysis (Gastropoda, Pseudoscorpionida, Hymenoptera other than Formicidae, and carrion from dead birds) and three groups undetected in the morphological random subsamples (Araneae, Diptera, and Lepidoptera). A similar situation was observed in Caragol (Fisher Exact Test, *p* = 0.6233), where the diet was dominated by Isopoda, Coleoptera, and Formicidae (Table 1) and in Na Guardia, where a very diverse diet was detected, with a small presence of each prey type in both samples (Fisher Exact Test, *p* = 0.2841). In the case of the Moltona Islet, molecular and morphological results were very different, because of a higher detection of groups using the molecular approach (Fisher Exact Test, *p* = 0.01125). This was also the case for Na Pelada Islet samples (Fisher Exact Test, *p* = 6.86 × 10^−5^).

Trophic diversity was significantly higher in molecular samples (Shannon index for morphological samples, H_sh_ = 2.239 ± 0.011, and for molecular samples, H_sh_ = 2.451 ± 0.006, *p* < 0.001; Simpson index for morphological samples, H_si_ = 0.837 ± 0.0005, and for molecular samples, H_si_ = 0.878 ± 0.0002, *p* < 0.001).

## 4. Discussion

Previous literature suggests that the use of metabarcoding allows for greater precision in prey identification, as well as the taxonomic identification of plant elements in the organism’s diet [12]. Moreover, a molecular approach may lead to an increase in the number of identifiable prey items [12,44]. In our study, numerous prey items were identified at the Species level, while others were only identified at the Order or Family level using morphological methods of diet analysis (see also [12]). This was particularly true for some groups, such as Diptera, Hemiptera, Lepidoptera, and Coleoptera (Table 1 and Table 2). Moreover, molecular analysis allowed the discovery of groups not detected using morphological analysis, such as Psocoptera, Embioptera, or Neuroptera. We showed that, overall, diversities determined by molecular techniques were significantly higher than those determined using similar sample sizes of fecal pellets.

Nevertheless, the study of diet using molecular approaches also holds some limitations and disadvantages, particularly with respect to traditional morphological methods. For example, the use of metabarcoding prevents the identification of cannibalism [44], which occurs frequently in small Mediterranean islets [9]. Additionally, current molecular techniques do not yet allow us to establish the quantitative contribution to the diet of each prey type [44]. They only provide us with a snapshot of the diet of a species—we cannot estimate its diversity, biomass contribution, prey size distribution, and other dietary descriptors.

Unlike other studies, we did not assemble a reference collection of plant species and potential prey in the populations under study; we also note that these identifications are not always reliable. Identified taxa sometimes belong to Species or Genera that are not present in the western Mediterranean or the Balearic Islands. Consequently, a *post hoc* analysis of these preliminary identifications was necessary, using taxonomic literature. In addition, an increase in the number of prey taxa identified through metabarcoding did not always occur. In most populations, we identified some additional prey using molecular methods, while other prey, even at the order level, were only identified through morphological observation of fecal pellets, and did not appear in the molecular samples. Hence, it is clear that the ideal scenario is to employ both methodologies as complementary approaches to describe the diet of a species [44].

Some molecular findings were difficult to interpret. In the Na Moltona Islet, the presence of DNA from the genus *Mus,* was detected in lizard scats. The presence of the House mouse (or the Algerian mouse, *Mus spretus*) on this coastal islet with a long history of human presence [45] cannot be ruled out. In fact, these species have been reported from islands with larger surface areas in the Balearics, but could be absent from most of the coastal islets. If this is the situation, we can posit that DNA from this small mammal was obtained from pellets of such scavengers as the Yellow-footed gull, *Larus michahellis* (see similar results in [12]).

Other results of the molecular analysis deserve our attention, such as the frequent consumption of several species of crustaceans. In many cases, these are terrestrial isopods, well-known from the diet of *P. lilfordi,* and present in morphologically analyzed samples [9]. Such is the case with the genus *Porcellio*. This is also the case with *Ligia italica*, a very common marine isopod in the diet of this species of lacertid lizards (see, for example, [9]). The significance of these findings, apart from the specific identification, is the confirmation of lizard foraging in shore areas and the frequent consumption of marine subsidies [12]. In the Caragol islet sample, we detected the presence of DNA of true marine crustaceans, particularly *Pachygrapsus marmoratus*, an extremely common decapod from the shore waters of the Balearic Islands [46], which had already been detected as carrion food of *P. lilfordi* from Aire Island [9]. This foraging behavior was confirmed by the presence of Cymodoceaceae DNA in samples from the same islet (Table 1). The only species of this seagrass present on the coast of Mallorca is *Cymodocea nodosa* [47]. In fact, some individual lizards seem prone to forage in shore areas. In Caragol Islet, from a single molecular fecal sample, we detected the presence of *P. marmoratus, L. italica,* and the seagrass *C. nodosa* (Table 1).

Regarding the consumption of some plant species, the molecular results must also be interpreted with caution and contrasted with morphological and ecological data. The supposed consumption of the genus *Pinus* has been detected in three populations where there are no pines, although these populations are close to the coast of Mallorca Island, an area with abundant pine forests. Thus, access to the fruit seems unlikely and the needles do not seem appropriate as a nutritive element [9]. This result poses a problem of a more general nature, i.e., the possibility that the detection of DNA from some plant species may be due to the adherence of pollen grains to the outer surface of some fecal pellets. In our samples, this possibility can be ruled out since the droppings were obtained directly from captured individuals. However, pollen grains of plant species not directly consumed by lizards may be present on the outer surfaces of other prey items [12]. It is important to note that the metabarcoding of plants cannot distinguish between the different parts of a plant species, such as its leaves, pollen, or fruit.

On Guardia and Pelada islets, DNA from the genus *Prunus*, which includes several species of fruit trees, was detected in feces. There are no species of this genus on either of the two islets. We suspect that its presence in our samples of lizard feces was due to the provision of fruit by visitors to the islets.

## 5. Conclusions

We can conclude that the application of molecular techniques in the study of lizard diets is extremely useful in combination with the traditional morphological analysis of feces. Using molecular evidence, we confirmed some direct observations of food consumption and found new taxa not detected using morphological analysis. Moreover, we were able to establish the specific identification of carrion, marine subsidies, and several common preys, such as ants. This study has the potential to be instructive by drawing the attention to new food items [48].

## Figures and Tables

**Table 1:** List of the identified diet taxa to the maximum resolution obtained in the diet composition of *Podarcis lilfordi* through molecular analysis. We show the number of fecal samples where each taxon was detected.

Phylum	Class	Order	Family	Genus	Species	Ses Bledes	Caragol	Na Guardia	Na Moltona	Na Pelada
**Arthropoda**										
	Arachnida	Araneae	Gnaphosidae			1				
		Pseudoscorpiones								1
		Trombidiformes						1		2
			Eriophyidae					1		
			Tydeidae			1				3
	Malacostraca	Decapoda	Grapsidae	*Pachygrapsus*	*marmoratus*		1			
		Isopoda				1	5	2	7	6
			Armadillidae	*Armadillo*	*officinalis*					1
			Halophilosciidae	*Halophiloscia*		2	1			4
				*Halophiloscia*	*couchii*				2	
			Ligiidae	*Ligia*	*italica*		3		2	
	Insecta					5				
		Archaeognatha	Machilidae						1	
		Coleoptera				1	3	1		1
			Anobiidae	*Gastrallus*		1	1			
			Curculionidae	*Tychius*					1	
		Diptera					1	1		
			Sarcophagidae	*Sarcophaga*		1				
			Syrphidae							1
		Embioptera						2		
			Oligotomidae	*Haploembia*					1	
		Hemiptera	Lygaeidae	*Nysius*						3
			Miridae							1
			Scutelleridae	*Odontoscelis*					1	
		Hymenoptera	Formicidae				1		1	
				*Messor*	*bouvieri*		2	1	1	
				*Pheidole*		6	4		5	
				*Plagiolepis*			1	1		
				*Tetramorium*	*semilaeve*				1	
		Lepidoptera	Crambidae	*Pyrausta*	*sanguinalis*	1				
			Psychidae					1		
			Pterophoridae	*Agdistis*	*meridionalis*					4
			Tortricidae			1				
				*Lobesia*						6
		Neuroptera	Coniopterydidae							1
		Psocoptera	Peripsocidae	*Peropsocus*					1	
			Trogiidae					1		2
				*Cerobasis*					3	
	Diplopoda	Julida	Julidae					2	3	1
**Chordata**										
	Mammalia	Rodentia	Muridae	*Mus*					2	
**Mollusca**										
	Gastropoda	Stylommatophora	Helicidae	*Theba*				1	1	
				*Theba*	*pisana*				2	
**Streptophyta**										
	Magnoliopsida	Alismatales					1			
		Apiales	Apiaceae				1			
		Caryophyllales				2	1	2	2	
			Amaranthaceae			2	2	2	4	5
				*Beta*	*vulgaris*	2			1	
				*Suaeda*		2	7	6	6	8
			Plumbaginaceae	*Limonium*		1	3	2	2	8
			Polygonaceae						1	
		Fabales	Fabaceae			1		2	2	1
				*Medicago*		2				
				*Prunus*				1		1
	Pinopsida	Pinales	Pinaceae	*Pinus*			2	1	2	

**Table 2:** List of the presence of identified diet taxa in morphological (morpho) and molecular analysis (DNA) of *Podarcis lilfordi* in the five populations under study. Taxa are grouped at the level of morphological identification (see more details in the text).

	Ses Bledes		Caragol		Na Guardia		Na Moltona		Na Pelada	
Taxon	Morpho	DNA	Morpho	DNA	Morpho	DNA	Morpho	DNA	Morpho	DNA
Gastropoda	1	0	1		0	1	5	1	1	0
Pseudoscorpionida	1	0							0	1
Araneae	0	1			1	0	0	2	2	0
Acarina					0	3			0	3
Isopoda	1	2	6	4	0	1	0	7	2	5
Crustacea			0	3			0	3	0	1
Diplopoda					1	2	1	3	0	1
Blattodea					1	0				
Isoptera							1	0		
Orthoptera		1								
Dermaptera		0					1	0		
Embioptera					0	2	0	1		
Homoptera		0							3	0
Heteroptera					3	0	0	1	0	3
Diptera	0	1	1	1	1	1				
Lepidoptera	0	2			0	1			0	6
Neuroptera	0	5							0	1
Coleoptera	1	2	8	4	4	1	4	1	3	1
Hymenoptera	1	0	2	0	1	0			3	0
Formicidae	7	6	8	5	3	1	6	4	6	0
Psocoptera					0	1	0	3	0	2
Archaeognatha							0	1		
Mammalia							0	1		
Aves	2	0								

## Data Availability

The data presented in this study are available on request from the corresponding author. Raw sequences are available in the Sequence Read Archive (SRA) database at NCBI under BioProject ID PRJNA703933.

## Appendix A

**Lists A1 and A2.** Additional list of references employed to check the validity of molecular identifications from metabarcoding, according to the taxonomic literature of prey and plants of Balearic Islands.

**Prey References**

- Alemany, A.; Leza, M.M.; Núñez, L.; Petro, B.; Closa, S.; Miranda, M.A. Estudio del impacto de los tratamientos contra la procesionaria del pino (*Thaumetopoea pityocampa*, Denn. y Schiff.) en Baleares. XXVI Grupo de Trabajo Fitosanitario, Govern de les Illes Balears, **2009**.
- Alonso-Zarazaga, M.A. Elenco sistemático de los Curculionoidea (Coleoptera) de la Península Ibérica e islas Baleares. *Boletín de la Sociedad Entomológica Aragonesa (S.E.A.)* **2018**, *63*, 3–44.
- Altaba, C.R. XXVII. Els caragols i llimacs terrestres (Mollusca: Gastropoda). In: Alcover, J.A.; Ballesteros, E.: Fornós, J.J. (Eds.), *Història Natural de l’Arxipèlag de Cabrera*; CSIC–Editorial Moll, Palma, Spain, **1993,** Monografies de la Societat d’Història Natural de les Balears, 2, pp. 409–426.
- Anonymous. *Balance fitosanitario–Baleares 2015*. Direcció General d’Agricultura I Ramaderia, Conselleria de medi Ambient, Agricultura i Pesca, Govern de les Illes Balears, Palma, Spain, **2015**.
- AntWiki. Last updated 01/0/2021. Available at https://www.antwiki.org/ [02/09/2021].
- Arbea, J.I. Los colémbolos de Aragón (Hexapoda: Collembola). *Catalogus de la entomofauna aragonesa* **2003**, *29*, 3–23.
- Arbea, J.I.; Jordana, R. Colémbolos de las Islas Baleares (Insecta, Collembola). *Redia* **1990**, *73*, 187–200.
- Bach, C.; Molero, R.; Gaju, M. Clase Insecta. Orden Microcoryphia. *Revista IDE@-SEA* **2015**, *38*, 1–12.
- Bächli, G.; Báez, M. Drosophilidae. In: *Catálogo de los Diptera de España, Portugal y Andorra (Insecta)*; Carles-Tolrá Hjorth-Andersen, M. (edit.) Monografías S.E.A., Zaragoza, Spain, **2002**; 8, 161–162.
- Bahillo de la Puebla, P.; López-Colón, J.I. Citas interesantes de cléridos de la Península Ibérica (Coleoptera, Cleridae). *Zoologica baetica* **1999**, *10*, 207–209.
- Bahillo de la Puebla, P.; López-Colón, J.I. El género *Opilo* Latreille, 1802 en la Península Ibérica (Coleoptera, Cleridae). *Boletín de la Asociación Española de Entomología* **2000**, 24 (1–2), 213–227.
- Baldizzone, G. Contributions à la connaissance des Coleophoridae. XLII. Sur quelques Coleophoridae d’Espagne (Seconde partie: Espèces nouvelles pour la Faune espagnole, ou peu connues). Nota lepidopterrologica **1986**, 9(1–2), 2–34.
- Baldock, D. A provisional list of the wasps and bees of Mallorca, Balearic Islands, Spain (Hymenoptera aculeata: Chrysidoidea, Scolioidea, Vespoidea, Apoidea). *Entomofauna* **2014**, *35*, 333–404.
- Baquero, E.; Jordana, R. Clase Collembola. Órdenes Poduromorpha, Entomobryomorpha, Neelipleona y Symphypleona. *Revista IDE@-SEA* **2015**, *36*, 1–11.
- Baank, R.A. Fauna Europaea: Gastropoda. **2013**, Fauna Europaea version 2017.06. https://fauna-eu.org [30/01/2021].
- Barrientos, J.A.; Febrer, B. Arañas (Arachnida, Araneae) de Menorca (Islas Baleares, España). 2: “Adenda et corrigenda”. Descripción de tres especies nuevas. *Revista Ibérica de Aracnología* **2018**, *33*, 39–51.
- Blanes-Dalmau, M.; Caballero-López, B.; Pujade-Villar, J. Estudi de les gales de la coŀlecció Vilarrúbia dipositada al Museu de Ciències Naturals de Barcelona. *Butlletí de la Institució Catalana d’Història Natural* **2017**, *81*, 137–173.
- Bohn, H. Revision of the *Loboptera* species of Spain (Blattaria: Blattellidae). *Entomologica Scandinavica* **1990**, *21*, 369–403.
- Bouaziz-Yahiatene, H.; Pfarrer, B.; Medjdoub-Bensaad, F.; Neubert, E. Revision of *Massylaea* Möllendorff, 1898 (Stylommatophora, Helicidae). *ZooKeys* **2017**, *694*, 109–133. https://doi.org/10.3897/zookeys.694.15001.
- Boxshall, G. Fauna Europaea: Isopoda, Porcellionidae, Fauna Europaea version 2017.06. https://fauna-eu.org [29/01/2021].
- Branco, V.V.; Morano E.; Cardoso, P. An update to the Iberian spider checklist (Araneae). *Zootaxa* **2019**, 4614 (2), 201–254.
- Cabanillas, D.; Parejo-Pulido, D. Primer registro de *Lamyctes (Lamyctes) emarginatus* (Newport, 1844) (Chilopoda: Lithobiomorpha: Henicopidae) en la Comunidad Autónoma de Extremadura y otras citas de la provincia de Badajoz (España). *Boletín de la Sociedad Entomológica Aragonesa* **2019**, *64*, 307–311.
- Carles-Tolrá, M.; Báez, M. Stratiomyidae. In: Carles-Tolrá Hjorth-Andersen, M. (coord.) *Catálogo de los Diptera de España, Portugal y Andorra (Insecta)*. Monografías S.E.A. **2002**, *8*, 113–114.
- Carles-Tolrá, M.; Báez, M. Therevidae. In: Carles-Tolrá Hjorth-Andersen, M. (edit.) *Catálogo de los Diptera de España, Portugal y Andorra (Insecta)*. Monografías S.E.A. **2002**, *8*, 117.
- Cartes, J.E.; Abelló, P.; Torres, P. The occurrence of *Hymenopenaeus debilis* (Decapoda: Aristeidae: Solenocerinae) in Mediterranean waters: A case of pseudopopulations of Atlantic origin? *Journal of the Marine Biological Association of the United Kingdom* **2000**, *80*, 549–550.
- Choi, E.H.; Hwang, U.W. First Record of Maritime Pseudoscorpion *Garypus japonicus* (Garypidae) from Korea. *Animal Systematics, Evolution and Diversity* **2009**, *25*, 261–264. https://doi.org/10.5635/KJSZ.2009.25.3.261.
- Chueca, L.J.; Madeira, M.J.; Gómez-Moliner, B.J. Biogeography of the land snail genus *Allognathus* (Helicidae): Middle Miocene colonization of the Balearic Islands. *Journal of Biogeography* **2015**, *42*, 1845–1857.
- Chueca, L.J.; Forés, M.; Gómez-Moliner, B.J. Actualización taxonómica y nomenclatural de las especies de *Xerocrassa* (Gastropoda: Geomitridae) endémicas de las islas Baleares. *Iberus* **2017**, *35*, 159–184.
- Cifuentes, J. Los isópodos terrestres de Galicia, España (Crustacea: Isopoda, Oniscidea). *Graellsia* **2019**, *75*, e098. https://doi.org/10.3989/graellsia.2019.v75.243.
- Cini, A.; Anfora, G.; Escudero-Colomar, L.A.; Grassi, A., Santosuosso, U.; Seljak, G.; Papini, A. Tracking the invasion of the alien fruit pest *Drosophila suzukii* in Europe. *Journal of Pest Science* **2014**, *87*, 559–566.
- Cobo, F.; Soriano, O.; Báez, M. Chironomidae. In: Carles-Tolrá Hjorth-Andersen, M. (coord.) *Catálogo de los Diptera de España, Portugal y Andorra (Insecta)*. Monografías S.E.A. **2002**, *8*, 35–44.
- Collingwood, C.A.; Yarrow, H.H. A survey of Iberian Formicidae (Hymenoptera). *Eos, Revista Española de Entomología* **1969**, *44*, 53–101.
- Cornara, D.; Garzo, E.; Morente, M.; Moreno, A.; Alba-Tercedor, J.; Fereres, A. EPG combined with micro-CT and video recording reveals new insights on the feeding behavior of *Philaenus spumarius*. *PLoS ONE* **2018**, *13*, e0199154. https://doi.org/10.1371/journal.pone.0199154.
- Costas, M.; López, T.; Vázquez, M.A. Checklist de Fauna Ibérica. Superfamilia Lygaeoidea Schilling, 1829 (Insecta: Heteroptera) en la península ibérica, islas Baleares e islas Canarias. In: Ramos, M.A. and Sánchez, M. (eds.) *Documentos Fauna Ibérica* **2018**, *7*, 1–29.
- Cruz-Suárez, A. Los *Halophilosciidae* Verhoeff, 1908 de la Península Ibérica e Islas Baleares (Isopoda: Oniscidea). *Boletín de la Asociación Española de Entomología* **1992**, *16*, 113–121.
- Cruz-Suárez, A. El género *Armadillidium* Brandt, 1833 en la Península Ibérica y Baleares (Isopoda, Oniscidea, Armadillidiidae). *Boletín de la Asociación Española de Entomología* **1993**, *17*, 155–181.
- Cruz, A. Redescripción de *Agabiformius obtusus* (Budde-Lund, 1909) y de *Armadillo hirsutus* Koch, 1856 (Isopoda: Oniscidea) de la Península Ibérica. *Butlletí de la Institució Catalana d’Història Natural* **1994**, *62*, 65–76.
- De Moraes, F.J.; McMurtry, J.A.; Denmark, H.A.; Campos, C.B. A revised catalog of the mite family Phytoseiidae. *Zootaxa* **2004**, *434*, 1–494.
- Delgado-Serra, S.; Viader, M.; Ruiz-Arrondo, I.; Miranda, M.A.; Barceló, C.; Bueno-Marí, R.; Hernández-Triana, L.M.; Miquel, M.; Lester, K.; Jurado-Rivera, J.A.; Paredes-Esquivel, C. Molecular Characterization of Mosquito Diversity in the Balearic Islands. *Journal of Medical Entomology* **2020**, *217*, 1–8. https://doi.org/10.1093/jme/tjaa217.
- De Lillo, E. Fauna Europaea: Eriophyidae. In Fauna Europaea: Actinotrichida. Magowski, W. **2003**, Fauna Europaea version 2017.06. https://fauna-eu.org [29/01/2021].
- Denux, O.; Zagatti, P. Coleoptera families other than Cerambycidae, Curculionidae *sensu lato*, Chrysomelidae *sensu lato* and Coccinelidae. Chapter 8.5. In *Alien terrestrial arthropods of Europe*. Roques, A.; Kenis, M.; Lees, D.; Lopez-Vaamonde, C.; Rabitsch, W.; Rasplus, J.Y.; Roy, D. (Eds.) *BioRisk* **2010**, *4*, 315–406. https://doi.org/10.3897/biorisk.4.61.
- Diéguez Fernández, J.M. Nuevas citas y catálogo de los Cantharidae y Dasytidae (Coleoptera) del área iberobalear. *Heteropterus Revista de Entomología* **2011**, *11*, 75–85.
- Docavo, A. Contribución al conocimiento de los Braconidae de España. I. Nuevos hallazgos de géneros y especies. *Entomophaga* **1962**, *7*, 343–348.
- Ebejer, M.J. Sorne Chloropidae (Diptera) from the Balearic Islands (Spain) with particular reference to Parc Natural de s’Albufera de Mallorca. *Bolletí de la Societat d’Història Natural de les Balears* **2006**, *49*, 173–184.
- Eidmann, H. Die Ameisenfauna der Balearen. *Zeitschrift für Morphologie und Ökologie der Tiere* **1926**, *6*, 694–742.
- Eidmann, H. Zur Kenntnis der Insektenfauna der balearischen Inseln. *Entomologische Mitteilungen* **1927**, 16: 24–37.
- Eiroa, E.; Báez, M. Tipulidae. In: Carles-Tolrá Hjorth-Andersen, M. (coord.) *Catálogo de los Diptera de España, Portugal y Andorra (Insecta)*. Monografías S.E.A. **2002**, *8*, 79–81.
- Ellis, W.N. Plant Parasites of Europe. Leafminers, galls and fungi. Available In https://bladmineerders.nl/ [27-01-2021].
- Español, F. De re entomològica Contribució a l’Entomologia de les illes de Cabrera i Foradada (Balears). *Butlletí de la Institució Catalana d’Història Natural* **1935**, *35*, 251–253.
- Español, F. Los Cléridos (Cleridae) de Cataluña e Islas Baleares (Col., Cleroidea). *Publicaciones del Instituto de Biología Aplicada* **1959**, *30*, 105–146.
- Español, F. *Coleoptera, Anobiidae*. *Fauna Ibérica, vol. 2*. Madrid: Museo Nacional de Ciencias Naturales. CSIC; Madrid, Spain **1992**, 192 pp.
- FAO FishFinder. Species Fact Sheets. *Pleoticus muelleri* (Bate, 1888). In FAO Fisheries Division [online]. Rome. [31/01/2021]. **2021**, Online at http://www.fao.org/fishery/species/3437/en.
- Faraji, F.; Ueckermann, E.A. A new species of *Mediolata* Canestrini from Spain (Acari: Stigmaeidae), re-description of *M. chanti* and a key to the known species of *Mediolata*. *Zootaxa* **2006**, *1151*, 27–39.
- Fet, V.; Soleglad, M.E. Morphology analysis supports presence of more than one species in the “*Euscorpius carpathicus*” complex (Scorpiones: Euscorpiidae). *Euscorpius, Occasional Publications in Scorpiology* **2002**, 3.
- Fischer S.; Patzner, R.A.; Müller, C.H.G.; Winkler, H.M. Studies on the ichthyofauna of the coastal waters of Ibiza (Balearic Islands, Spain). *Rostocker Meeresbiologische Beiträge* **2007**, *18*, 30–62.
- Gabarra, R.; Arnó, J.; Riudavets, J. *Drosophila suzukii*: Biología y ecología. *PHYTOMA España* **2015**, *269*, 12–13.
- Gadea, E. Nematofauna muscícola. In *Història Natural de l’Arxipèlag de Cabrera*; Alcover, J.A.; Ballesteros, E.; Fornós, J.J. (Eds.); CSIC–Editorial Moll, Palma, Spain, Monografies de la Societat d’Història Natural de les Balears **1993**, *2*, 269–272.
- Gangwere, S.K.; Llorente, V. Distribution and habits of the Orthoptera (*sens. lat*.) of the Balearic Islands (Spain). *Eos* **1992**, *68*, 51–87.
- Gantenbein, B.; Soleglad, M.E.; Fet, V. *Euscorpius balearicus* Caporiacco, 1950, stat. nov. (Scorpiones:Euscorpiidae): Molecular (allozymes and mtDNA) and morphological evidence for an endemic Balearic Islands species. *Organisms Diversity and Evolution* **2001**, *1*, 301–320.
- García, L. *Halophiloscia ischiana* Verhoeff, 1933, un isòpode terrestre nou per a la fauna de Mallorca. *Aubaïna* **2000**, *2*, 18.
- García, L. *Armadillidium cruzi*. In *Bioatles*. Palma: Servei de Protecció d’Especies, Conselleria de Medi Ambient, Palma, Spain, **2006**.
- García, L.; Cruz, A. XIX. Els isopòdes terrestres (Crustacea: Isopoda: Oniscidea). In, *Història Natural de l’Arxipèlag de Cabrera*; Alcover, J.A.; Ballesteros, E.; Fornós, J.J. (Eds.); CSIC–Editorial Moll, Palma, Spain, Monografies de la Societat d’Història Natural de les Balears **1993**, *2*, 323–332.
- García, L.; Cruz, A. Els isòpodes terrestres (Crustacea: Isopoda: Oniscidea) de les iIles Balears: Catàleg d’especies. *Bolletí de la Societat d’Història Natural de les Baleares* **1996**, *39*, 77–99.
- García, L.; Gross, A.; Riddiford, N. *Armadillidium album*, un isopode terrestre nou per a la fauna balear (Isopoda, Crinocheta, Armadillidiidae). *Bolletí de la Societat d’Història Natural de les Baleares* **2004**, *46*, 91–94.
- García-Barros, E.; Romo, H.; Sarto i Monteys, V.; Munguira, M.L.; Baixeras, J.; Vives Moreno, A.; Yela García, J.L. Clase Insecta, Orden Lepidoptera. *Revista IDE@-SEA* **2006**, 65: 1–21.
- García-Romera, C.; Báez, M. Phoridae. In *Catálogo de los Diptera de España, Portugal y Andorra (Insecta);* Carles-Tolrá Hjorth-Andersen, M. (coord.). Monografías S.E.A. **2002**, *8*, 125–129.
- García-Romera, C.; Barrientos, J.A. La fauna de Phoridae (Diptera) en el parque natural del Montseny (Cataluña, España). Citas nuevas para la Península Ibérica. *Boletín de la Sociedad Entomológica Aragonesa*, **2014**, *54*, 237–261.
- Gasull, L. Las *Helicella* (*Xeroplexa*) de Baleares, Gasteropoda Pulmonata. *Boletín de la Sociedad de Historia Natural de Baleares* **1964**, *10*, 3–70.
- Gasull, L. Algunos moluscos terrestres y de agua dulce de Baleares. *Boletín de la Sociedad de Historia Natural de Baleares* **1965**, *11*, 7–161.
- GBIF.org. GBIF: The Global Biodiversity Information Facility. Available in https://www.gbif.org [22-01-2021].
- Germann, C.; Torres, J.L.; Borovec, R. Confirmative records of *Trachyphloeus nodipennis* Chevrolat, 1860 for the Iberian Peninsula (Coleoptera: Curculionidae: Entiminae) with a key to the Spanish species of the nominal subgenus. *Boletín de la SAE* **2017**, *27*, 23–28.
- Gnezdilov, V.M.; Holzinger, W.E.; Wilson, M.R. The Western Palaearctic Issidae (Hemiptera, Fulgoroidea): An Illustrated Checklist and Key to Genera and Subgenera. *Proceedings of the Zoological Institute RAS* **2014**, 318 (1), 5–118.
- Goldarazena, A. Clase Insecta, Orden Thysanoptera. *Revista IDE@-SEA* **2015**, *52*, 1–20.
- Gómez, K. Citas nuevas o interesantes de hormigas (Hymenoptera: Formicidae) para la isla de Mallorca (Baleares, España). *Boletín de la Sociedad Entomológica Aragonesa* **2004**, *34*, 107–108.
- Gómez, K.; Espadaler, X. *La hormiga argentina (Linepithema humile) en las Islas Baleares. Listado preliminar de las Hormigas de las Islas Baleares*. Documentos Técnicos de Conservación. Conselleria de Medi Ambient. Govern de les Illes Balears **2005**, *13*, 68 pp.
- Gómez, K.; Espadaler, X. Exotic ants (Hymenoptera: Formicidae) in the Balearic Islands. *Myrmecologische Nachrichten* **2006**, *8*, 225–233.
- González, M.A.; Terra, L.S.W.; García de Jalón, D.; Cobo, F. *Lista faunística y bibliográfica de los Tricópteros (Trichoptera) de la Península Ibérica e Islas Baleares*. Listas de la Flora y Fauna de las aguas continentales de la Península Ibérica, **1992**, 11. Madrid: Asociación Española de Limnología. 200pp.
- González Peña, C.F.; Vives i Noguera, E.; de Sousa Zuzarte, A.J. *Nuevo catálogo de los Cerambycidae (Coleoptera) de la Península Ibérica, islas Baleares e islas atlánticas: Canarias, Açores y Madeira*. Monografías S.E.A. **2007**, *12*, 1–211.
- Govern Balear. *La procesionaria del pino*. Conselleria d’Agricultura i Pesca, Direcció General de Producció i Industries Agràries, Palma, Spain, **2011.**
- Gravestein, W.H. Twaalf nieuwe Hemiptera Heteroptera voor de fauna van Mallorca. *Entomologische Berichten* **1969**, *29*, 156–158.
- Gravestein, W.H. Hemiptera Heteroptera new to the Baleares, in particular to the Island of Mallorca. *Entomologische Berichten* **1978**, *38*, 37–39.
- Harbach, R.E. Genus *Acartomyia* Theobald, 1903. Mosquito Taxonomic Inventory. Available in http://mosquito-taxonomic-inventory.info/genus-acartomyia-theobald-1903-0#overlay-context=genus-acartomyia-theobald-1903-0 [05/02/2021].
- Harvey, M.S.; Hillyer, M.J.; Carvajal, J.I.; Huey, J.A. Supralittoral pseudoscorpions of the genus *Garypus* (Pseudoscorpiones: Garypidae) from the Indo-West Pacific region, with a review of the subfamily classification of Garypidae. *Invertebrate Systematics* **2020**, *34*, 34–87. https://doi.org/10.1071/IS19029.
- Heckman, C.W. *Neuroptera (Including Megaloptera)*., Springer, Switzerland, **2017** 621pp.
- Henry, T.J.; Dellapé, P.M.; Scudder, G.G.E. Resurrection of the Genera *Crophius* Stål and *Mayana* Distant from Synonymy Under Anomaloptera Amyot and Serville, Description of a New Genus, and a Key to the New World Oxycarenid Genera (Hemiptera: Heteroptera: Oxycarenidae). *Proceedings- Entomological Society of Washington* **2015**, *117*, 367–380.
- Hodkinson, I.D.; Hollis, D. The psyllids (Homoptera: Psylloidea) of Mallorca. *Entomologica Scandinavica* **1981**, *12*, 65–77.
- Hurtado L.A.; Lee E.J.; Mateos M.; Taiti S. Global Diversification at the Harsh Sea-Land Interface: Mitochondrial Phylogeny of the Supralittoral Isopod Genus *Tylos* (Tylidae, Oniscidea). *PLoS ONE* **2014**, *9*, e94081. https://doi.org/10.1371/journal.pone.0094081.
- Iaciofano, D.; Lo Brutto, S. Re-description of *Orchestia stephenseni* Cecchini, 1928: Designation of neotype and senior synonym to *Orchestia constricta* A. Costa, 1853 (Crustacea: Amphipoda: Talitridae) by Reversal of Precedence. *Zootaxa* **2016**, 4150 (1), 40–60.
- Iberfauna. IBERFAUNA. El Banco de Datos de la Fauna Ibérica. Museo Nacional de Ciencias Naturales (CSIC). Available in http://iberfauna.mncn.csic.es/showficha.aspx?rank=T&idtax=9657 [27/01/2021].
- Jaramillo, E.; Cifuentes, S.; Duarte, C.; Contreras, H. Relationships between bioturbation by *Tylos spinulosus* (Crustacea, Isopoda) and its distribution on sandy beaches of north-central Chile. *Marine Ecology* **2008**, *29*, 37–42.
- Jordana, R.; Arbea, J.I. Clave de identificación de los géneros de colémbolos de España (Insecta: Collembola). *Publicaciones de Biología de la Universidad de Navarra, Serie Zoológica* **1989**, *19*, 1–16.
- Jurado-Rivera, J.A.; Álvarez, G.; Caro, J.A.; Juan, C.; Pons, J.; Jaume, D. Molecular systematics of *Haploginglymus*, a genus of subterranean amphipods endemic to the Iberian Peninsula (Amphipoda: Niphargidae). *Contributions to Zoology* **2017**, 86 (3), 239–260.
- Karsholt, O.; Nieukerken, E.J. van. Lepidoptera. Fauna Europaea version 2017.06, https://fauna-eu.org [29/01/2021].
- Kawachino, Y. Spawning record of *Pythia cecillei* (Philippi, 1847). *Transactions of the Nagasaki Biological Society* **2015**, *76*, 62–66.
- Kazantsev, S. Fauna Europaea: *Malthodes*. In: Alonso-Zarazaga, M.A. **2013**. Fauna Europaea: Coleoptera, Cantharidae. Fauna Europaea version 2017.06. https://fauna-eu.org [29/01/2021].
- Kennedy, M.; Spencer, H.G. Classification of the cormorants of the world. *Molecular Phylogenetics and Evolution* **2014**, *79*, 249–257.
- Klimov, P.B.; OConnor, B.; Ochoa, R.; Bauchan, G.R.; Redford, A.J.; Scher, J. Bee Mite ID: Bee-Associated Mite Genera of the World. USDA APHIS Identification Technology Program (ITP), Fort Collins, CO. [24-01-2021] Available in http://idtools.org/id/mites/beemites/index.php.
- Kuschel, G. Curculionoidea (weevils) of New Caledonia and Vanuatu: Ancestral families and some Curculionidae. In:, *Zoologia Neocaledonica 6. Biodiversity studies in New Caledonia;* Grandcolas, P. (ed.); *Mémoires du Muséum National d’Histoire Naturelle* **2006**, *197*, 99–249.
- Lomnicki, J. Une contribution à la connaissance de la faune des fourmis des îles Baléares. *Polskie Pismo Entomologiczne-Bulletin Entomologique de la Pologne* **1925**, *4*, 1–3.
- Lowry, J.K.; Myers, A.A. New genera of Talitridae in the revised Superfamily Talitroidea Bulycheva 1957 (Crustacea, Amphipoda, Senticaudata). *Zootaxa* **2019**, 4553 (1), 1–100.
- Lucena-Moya, P.; Abraín, R.; Pardo, I.; Hermida, B.; Domínguez, M. Invertebrate species list of coastal lagoons in the Balearic Islands. *Transitional Waters Bulletin* **2010**, 4 (1), 1–11.
- Lundqvist, L. Fauna Europaea: Acari, Mesostigmata. **2013**, Fauna Europaea version 2017.06. https://fauna-eu.org [29/01/2021].
- Mahnert, V. XXII. Els pseudoscorpins (Arachnida, Pseudoscorpiones). In, *Història Natural de l’Arxipèlag de Cabrera*, Alcover, J.A., Ballesteros, E. and Fornós, J.J. (Eds.); CSIC–Editorial Moll, Palma, Spain; Monografies de la Societat d’Història Natural de les Balears **1993**, *2*, 355–360.
- Malicky, L.H. Beschreibungen von neuen mediterranen Köcherfliegen und Bemerkungen zu bekannten (Trichoptera). Zeitschrift der Arbeitsgemeinschaft Österreichischer Entomologen **1980**, *32*, 1–17.
- Malo, J.; García-Avilés, J. Contribución al conocimiento de los quironómidos (Diptera, Chironomidae) de las Islas Baleares. *Zoologica Baetica* **1999**, *10*, 211–214.
- Marcos-García, M.A.; Rojo, S.; Pérez-Bañón, C. Syrphidae. In: *Catálogo de los Diptera de España, Portugal y Andorra (Insecta)*. Carles-Tolrá Hjorth-Andersen, M. (coord.); Monografías S.E.A. **2002**, *8*, 132–136.
- Martín, J.L.; Izquierdo, I.; Oromí, P. The genus *Loboptera* in the Canary Islands; description of five new hypogean species. *Vieraea* **1999**, *27*, 255–286.
- Martínez-Fernández, J.C. Un nuevo representante del género *Blaps* Fabricius, 1775 de la Península Ibérica: *Blaps tichyi* n. sp. (Coleoptera, Tenebrionidae). *Boletín de la Sociedad Entomológica Aragonesa* **2010**, *47*, 181–185.
- Mastrantonio, V.; Porretta, D.; Bellini, R.; Nascetti, G.; Urbanelli, S. Molecular Systematics and Origin of the Mediterranean Sea Rock-Pool Mosquitoes of the *Aedes mariae* (Diptera: Culicidae) Complex. *Annals of the Entomological Society of America* **2015**, 108: 593–599. https://doi.org/10.1093/aesa/sav031.
- Mauriés, J.P.; Vicente, M.C. Miriápodos de Baleares. *Boletín de la Sociedad de Historia Natural de Baleares* **1976**, *21*, 33–46.
- Meliá, A. Contribución al conocimiento de los pulgones (*Homoptera, aphidoidea*) sobre plantas agrícolas y forestales en España. *Boletín de sanidad vegetal. Plagas* **1986**, *12*, 335–342.
- Mendoza-Roldan, J.A.; Colella, V.; Lia, R.P.; Nguyen, V.L.; Barros-Battesti, D.M.; Iatta, R.; Dantas-Torres, F.; Otranto, D. *Borrelia burgdorferi* (sensu lato) in ectoparasites and reptiles in southern Italy. *Parasites & Vectors* **2019**, *12*, 35 https://doi.org/10.1186/s13071-019-3286-1.
- Menozzi, C.C. Zur Kenntnis der Ameisenfauna der Balearen. Zoologische Anzeiger **1926**, 66(7–8), 180–182.
- Michalska, K.; Skoracka, A.; Navia, D.; Amrine, J.W. Behavioural studies on eriophyoid mites: An overview. *Experimental and Applied Acarology* **2009**, *51*, 31–59. https://doi.org/10.1007/s10493-009-9319-2.
- Miranda, M.A.; López-Mercadal, J.; Tugores, M.A.; Delgado, S.; Seguí, G.; Lalucat, J.; Gomila, M.; Ruíz, M.; Lester, K.; Kenyon, D.M.; Paredes-Esquivel, C. Recogida de datos e información en las Islas Baleares sobre la biología de vectores de *Xylella fastidiosa*. *XLIX Foro INIA.* **2019**, *Xylella fastidiosa en el contexto del cambio climático*.
- Monserrat, V.J. Catálogo de los Neurópteros de Baleares con nuevos datos sobre su fauna (Insecta, Neuroptera). *Bolletí de la Societat d’Història Natural de les Baleares* **2005**, *48*, 71–85.
- Monserrat, V.J. Los crisópidos de la Península Ibérica y Baleares (Insecta, Neuropterida, Neuroptera: Chrysopidae). *Graellsia* **2016a**, 72, e037. https://doi.org/10.3989/graellsia.2016.v72.143.
- Monserrat, V.J. Los coniopterígidos de la Península Ibérica e Islas Baleares (Insecta: Neuropterida, Neuroptera: Coniopterygidae). *Graellsia* **2916b**, 72, e047. http://dx.doi.org/10.3989/graellsia.2016.v72.157.
- Monserrat, V.J.; Acevedo, F.; Pantaleoni, R.A. Nuevos datos sobre algunas especies de crisópidos de la Península Ibérica, Islas Baleares e Islas Canarias (Insecta, Neuroptera, Chrysopidae). *Graellsia*, **2014**, *70*, e002;. https://doi.org/10.3989/graellsia.2014.v70.100.
- Montesanto, G.; Deidun, A.; Sciberras, A.; Sciberra, J.; Lombardo, B.M. Current distribution of two species of *Tylos* (Isopoda: Oniscidea) in the central Mediterranean and the influence of beach sand grain-size parameters. *Journal of Crustacean Biology* **2014**, *34*, 47–53. https://doi.org/10.1163/1937240X-00002206.
- Moraza, M.L.; Irwin, N.R.; Godinho, R.; Baird, S.J.E.; Bellocq, J.G. A new species of *Ophionyssus* Mégnin (Acari: Mesostigmata: Macronyssidae) parasitic on *Lacerta schreiberi* Bedriaga (Reptilia: Lacertidae) from the Iberian Peninsula, and a world key to species. *Zootaxa* **2009**, *2007*, 58–68.
- Moreno, A.G. Orden Astigmata. *Revista IDE@-SEA* **2015**, *15*, 1–19.
- Myers, A.A.; Lowry, J.K. A revision of the genus *Orchestia* Leach, 1814 with the reinstatement of *O. inaequalipes* (K.H. Barnard, 1951), the designation of a neotype for *Orchestia gammarellus* (Pallas, 1776) and the description of three new species (Crustacea: Amphipoda: Talitridae: Talitrinae). *Zootaxa* **2020**, 4808 (2): 201–250.
- Nentwig, W.; Blick, T.; Bosmans, R.; Gloor, D.; Hänggi, A.; Kropf, C. Spiders of Europe. Version 01.2021. Online at https://www.araneae.nmbe.ch, [22-01-2021]. https://doi.org/10.24436/1.
- Núñez, L. *Plagas de frondosas en las Illes Balears*. Servicio de Sanidad Forestal, Govern de les Illes Balears. Palma, Spain.
- OConnor; B.M. Evolutionary ecology of Astigmatid mites. *Annual Review of Entomology* **1982**, *27*, 385–409.
- Oosterbroek, P. Notes on western Palaearctic species of the *Tipula* (*Yamatotipula*) *lateralis* group, with the description of a new species from Turkey (Diptera: Tipulidae). *European Journal of Entomology* **1994**, *91*, 429–435.
- Ouvrard, D. Psyl’list-The World Psylloidea Database; Available in http://www.hemiptera-databases.com/psyllist [08/12/2020]. https://doi.org/10.5519/0029634.
- Palau, J.M. Algunas consideraciones sobre los embiópteros de Mallorca y, en especial, sobre el género *Haploembia* Verh. *Bolletí de la Societat d’Història Natural de les Balears* **1956**, *2*, 23–25.
- Palau, J.M. Pequeño catálogo de hemípteros heterópteros de Mallorca. *Boletín de la Sociedad de Historia Natural de Baleares* **1959**, *5*, 7–11.
- Palmer, M.; Petitpierre, E. XXVI. Els coleòpters de Cabrera: Llista faunística i perspectives d’estudi. In *Història Natural de l’Arxipèlag de Cabrera*; Alcover, J.A.; Ballesteros, E.; Fornós, J.J. (Eds.); CSIC–Editorial Moll, Monografies de la Societat d’Història Natural de les Balears **1993**, *2*, 383–407.
- Pape, T.; González-Mora, D.; Peris, S.V.; Báez, M. Sarcophagidae. In *Catálogo de los Diptera de España, Portugal y Andorra (Insecta)*; Carles-Tolrá Hjorth-Andersen, M. (coord.); Monografías S.E.A. **2002**, *8*, 218–221.
- Peña, L.E. Nuevas especies del género *Psammetichus* Latr., (Coleoptera-Tenebrionidae) para Chile y Perú. *Revista Chilena de Entomología* **1973**, *7*, 137–144.
- Perera, A.; Maia, J.P.M.C.; Jorge, F.; Harris, D.J. Molecular screening of nematodes in lacertid lizards from the Iberian Peninsula and Balearic Islands using 18S rRNA sequences. *Journal of Helminthology* **2012**, *87*, 189–194. https://doi.org/10.1017/S0022149x12000181.
- Pérez-Íñigo, C. jr. *Acari Oribatei, Poronota*. *Fauna Ibérica, vol. 3*, Museo Nacional de Ciencias Naturales. CSIC, Madrid, Spain, **1993**.
- Petitpierre, E.; Sacarés, A.; Jurado-Rivera, J.A. Updated checklist of Balearic leaf beetles (Coleoptera: Chrysomelidae). *Zootaxa* **2017**, 4272 (2), 151–177.
- Pons, G.X. XX. Estudi preliminar sobre la fauna d’aranèids (Arachnida, Araneae). In, *Història Natural de l’Arxipèlag de Cabrera*; Alcover, J.A.; Ballesteros, E.; Fornós, J.J. (Eds); CSIC–Editorial Moll, Monografies de la Societat d’Història Natural de les Balears **1993**, *2*, 333–350.
- Pons, G.X. Noves dades biogeografiques i taxonomiques sobre els escorpins (Arachnida; Scorpiones: Euscorpiidae) de les Illes Balears. *Bolletí de la Societat d’Història Natural de les Baleares* **2001**, *44*, 103–109.
- Pons, G.X.; Palmer, M. *Fauna endèmica de les illes Balears*. Palma (Spain): Institut d’Estudis Baleàrics, Conselleria d’Obres Públiques, Ordenació del Territori i Medi Ambient (Dir. Gen. Medi Ambient). Societat d’Història Natural de les Balears. Palma, Spain, **1996**, 307pp.
- Pons, G.X; Palmer, M. Invertebrats endemics i Illes: (Tenebrionidae i Araneae) introduccions i extincions aIs illots de Cabrera (Illes BaIears). In: Alcover, J.A. (coord.), *Ecologia de les illes*. Mon. Soc. Hist. Nat. Balears 6/Mon. Inst. Est. Bal. **1999**, *66*, 105–122. Palma de Mallorca, Spain.
- Pons, G.X.; Vadell, M. Biospeleologia de les cavitats de les Illes Balears: Invertebrats terrestres. *Endins* **2011**, *35*, 241–256.
- Pons, G.X.; Jaume, D.; Damians, J. Fauna cavernícola de Mallorca/Cavernicolous Fauna of Mallorca. *Endins* **1995**, *20*, 125–144.
- Pons, G.X.; Palmer, M.; García, Ll. Isópodos terrestres (Isopoda, Oniscidea) de las Islas Chafarinas (N África, Mediterráneo Occidental). *Bolletí de la Societat d’Història Natural de les Baleares* **1999**, *42*, 139–146.
- Pont, A.C.; Báez, M. Muscidae. In: Carles-Tolrá Hjorth-Andersen, M. (coord.) *Catálogo de los Diptera de España, Portugal y Andorra (Insecta)*. Monografías S.E.A. **2002**, *8*, 210–214.
- Redondo, V.M.; Gastón, F.J.; Gimeno, R. *Geometridae Ibericae*; Apollo Books; Stenstrup; Denmark, **2009**, 360p.
- Remaudiére, G.; Nieto, J.M.; Mier, M.P. Nuevas aportaciones al conocimiento de la fauna española de pulgones (*Hom. Aphidoidea*). *Boletín de la Asociación Española de Entomología* **1986**, *10*, 313–333.
- Requena, E. Noves dades sobre la distribució del genere *Agdistis* Hübner, [1825], a Catalunya (Lepidoptera: Pterophoridae). *Butlleti-Societat Catalana de Lepidopterologia* **1999**, *84*, 9–16.
- Requena, E. Aproximació a la fauna dels gelèquids de Catalunya i Balears (Lepidoptera: Gelechiidae). *Treballs de la Societat Catalana de Lepidopterologia* **2009**, *16*, 5–77.
- Ribera, I.; Melic, A. Clase Insecta. Orden Neuroptera s.s. (Planipennia). *Revista IDE@-SEA*, **2015**, *58*, 1–12.
- Ribes, J. Hemípteros de Mallorca. *Publicaciones del Instituto de Biología Aplicada* **1965**, *39*, 71–95.
- Ribes, J. XXIII. Els heteròpters. In *Història Natural de l’Arxipèlag de Cabrera*. Alcover, J.A.; Ballesteros, E.; Fornós, J.J. (Eds.); CSIC–Editorial Moll, Monografies de la Societat d’Història Natural de les Balears **1993**, *2*, 361–364.
- Robertson, P.L. A revision of the genus *Tyrophagus*, with a discussion on its taxonomic position in the Acarina. *Australian Journal of Zoology* **1959**, *7*, 146–181.
- Roca, V.; Hornero, M.J. *Strongyloides ophiusensis* sp. n. (Nematoda: Strongyloididae), parasite of an insular lizard, *Podarcis pityusensis* (Sauria: Lacertidae). *Folia Parasitologica* **1992**, *39*, 369–373.
- Roca-Cusachs, M.; Goula, M.; Prieto, F.; Pérez, J. Checklist de Fauna Ibérica. Superfamilias Aradoidea, Coreoidea y Pyrrhocoroidea (Insecta: Heteroptera) en la península ibérica, islas Baleares e islas Canarias. In: Ramos, M.A. and Sánchez, M. (eds.). *Documentos Fauna Ibérica* **2018**, 6. Museo Nacional de Ciencias Naturales, CSIC. Madrid. 14 pp.
- Rozkosny, R. Fauna Europaea: Stratiomyidae. In Fauna Europaea: Diptera, Brachycera; Pape, T.; Beuk, P. Fauna Europaea version 2017.06. https://fauna-eu.org [29/01/2021].
- Schmalfuss, H. World catalog of terrestrial isopods (Isopoda: Oniscidea). *Stuttgarter Beiträge zur Naturkunde* **2003**, *Serie A*, 654: 1–341.
- Schubart, C.D.; Cuesta, J.A.; Felder, D.L. Phylogeography of *Pachygrapsus transversus* (Gibbes, 1850): The effect of the American continent and the Atlantic Ocean as gene flow barriers and recognition of *Pachygrapsus socius* Stimpson 1871 as a valid species. *Nauplius* **2005**, *13*, 99–113.
- Seco, M.V.; Mier, M.P. Contribuciones al conocimiento de los pulgones (*Hom., Aphidoidea*) de las Islas Baleares. 1. Introducción y afidofauna de Mallorca. *Bolletí de la Societat d’Història Natural de les Balears* **1986**, *30*, 5–17.
- Seifert, B.; d’Eustacchio, D.; Kaufmann, B.; Centorame, M.; Lorite, P.; Modica, M.V. Four species within the supercolonial ants of the *Tapinoma nigerrimum* complex revealed by integrative taxonomy (Hymenoptera: Formicidae). *Myrmecological News* **2017**, *24*, 123–144.
- Siddiqi M.R. *Tylenchida. Parasites of plants and insects*., 2^nd^ ed. CABI Publishing. **2000,** 848 pp.
- Skuhravá, M. and Skuhravý, V. Gall midges (Cecidomyiidae, Diptera) of Mallorca (Balearic Islands, Spain). *Boletín de la Asociación Española de Entomología* **2004**, 28 (1–2), 105–119.
- Skuhravá, M.; Skuhravý, V. Species richness of gall midges (Diptera: Cecidomyiidae) in Europe (West Palaearctic): Biogeography and coevolution with host plants. *Acta Societatis Zoologicae Bohemicae* **2009**, *73*, 87–156.
- Skuhravá, M.; Blasco-Zumeta, J.; Pujade-Villar, J. Cecidomyiidae. In *Catálogo de los Diptera de España, Portugal y Andorra (Insecta)*; Carles-Tolrá Hjorth-Andersen, M. (coord.); Monografías S.E.A. **2002**, *8*, 21–25.
- Sláma, P.M.; Berger, P. Contribution to the knowledge of the genus *Nathrius* Brèthes, 1916, with the description of *N. cypericus* n. sp. from Cyprus (Coleoptera: Cerambycidae). *Biocosme Mésogéen* **2006**, *23*, 55–65.
- Soghigian, J.; Andreadis, T.G.; Livdahl, T.P. From ground pools to treeholes: Convergent evolution of habitat and phenotype in *Aedes* mosquitoes. *BMC Evolutionary Biology* **2017**, 17: 262. https://doi.org/10.1186/s12862-017-1092-y.
- Soler-Membrives, A.; Munilla, T. PYCNOIB: Biodiversity and Biogeography of Iberian Pycnogonids. *PLoS ONE* **2015**, *10*, e0120818. https://doi.org/10.1371/journal.pone.0120818.
- Spelda, J. Clase Diplopoda, Orden Julida. *Revista IDE@-SEA* **2015**, *27*, 1–18.
- Stehlík, J.L.; Kment, P. *Antilochus* (*Neaeretus*) *pterobrachys* sp. nov. and the correct name of the subgenus *Afroantilochus* (Hemiptera: Heteroptera: Pyrrhocoridae). *Acta Entomologica Musei Nationalis Pragae* **2011**, *51*, 49–53.
- Stock, J.H. On the identity of *Porrassia mallorquensis* Marcus, 1912, an amphipod supposedly endemic in Mallorca. *Crustaceana* **1976**, 30 (1), 110–111.
- Subías, L.S.; Shtanchaeva, U.Y.; Arillo, A. Oribátidos (Acari, Oribatida) de España peninsular e Islas Baleares. Distribución. *Monografías electrónicas S.E.A.* **2013**, 5. Sociedad Entomológica Aragonesa.
- Teruel, R.; Melic, A. Orden Scorpiones. *Revista IDE@*-SEA **2015**, *18*, 1–17.
- Thanou, E.; Sponza, S.; Nelson, E.J.; Perry, A.; Wanless, S.; Daunt, F.; Cavers, S. Genetic structure in the European endemic seabird, *Phalacrocorax aristotelis*, shaped by a complex interaction of historical and contemporary, physical and nonphysical drivers. *Molecular Ecology* **2017**, *26*, 2796–2811.
- Torres-Vila, L.M.; McMinn, M.; Rodríguez-Molina, A.; Rodríguez-Molina, M.C. Primera cita de *Lobesia botrana* Den. et Schiff. (Lepidoptera: Tortricidae) en la isla de Cabrera, Islas Baleares. *Bolletí de la Societat d’Història Natural de les Balears* **2006**, *49*, 45–49.
- Vadell, M.; Pons, G.X. Aportaciones al conocimiento de los quilópodos (Chilopoda; Geophilomorpha) de la Serra de na Burguesa (Mallorca, Islas Baleares). *Bolletí de la Societat d’Història Natural de les Balears* **2009**, *52*, 169–182.
- Vadell, M.; Zaragoza, J.A. Estudio preliminar de la fauna invertebrada terreste de la Cova des Coll (Felanitx, Mallorca). *Endins* **2005**, *27*, 187–204.
- Vallhonrat, F. Aportació a la fauna de geomètrids de les illes Balears (Lepidoptera: Geometridae). *Butlletí-Societat Catalana de Lepidopterologia* **2004**, *93*, 43–51.
- Vallhonrat, F.; Pérez, J.J.; Requena, E. Heteròcers nous o interessants de les illes Balears (Lepidoptera). *Butlletí-Societat Catalana de Lepidopterologia* **2011**, *102*, 67–72.
- Vicens, P. Primer cens hivernal de corb marí gros *Phalacrocorax carbo* a les zones de colgada a Balears. *Anuari Ornitològic de les Balears* **2012**, *27*, 15–21.
- Viejo, J.L.; González, J.; Gómez, C. Biodiversidad de lepidópteros en relación con sus hábitats, formaciones vegetales y flora de Las Marismillas (Parque Nacional de Doñana, Huelva, Sur de España). Resultados preliminares. *Boletín de la Real Sociedad Española de Historia Natural, Sección biológica* **2014**, *108*, 79–101.
- Wang, M.; Zhang, Y.; Bourgoin, T. Planthopper family Issidae (Insecta: Hemiptera: Fulgoromorpha): Linking molecular phylogeny with classification. *Molecular Phylogenetics and Evolution* **2016**, *105*, 224–234.
- Wheeler, W.M. Ants of the Balearic Islands. *Folia Myrmecologica et Termitologica* **1926**, *1*, 1–6.
- Wirth, S. Description of a new species, *Bonomoia opuntiae* n. sp. (Histiostomatidae, Astigmata), with observations on the function of its eyes. *Acarologia* **2005**, XLV, 4, 303–319.
- World Spider Catalog. Version 22.0. Natural History Museum Bern, online at http://wsc.nmbe.ch [22-01-2021]. https://doi.org/10.24436/2.
- Wulcan, J.M.; Dennis, M.M.; Ketzis, J.K.; Bevelock, T.J.; Verocai, G.G. *Strongyloides* spp. in cats: A review of the literature and the first report of zoonotic *Strongyloides stercoralis* in colonic epithelial nodular hyperplasia in cats. *Parasites Vectors* **2019**, *12*, 349.
- WWF España. Informe del Inventario: MAL028-S’Albufera de Mallorca. EsIsWet-Base de datos de los humedales insulares españoles. Updated: 05.**2020**. Online at: https://www.humedalesdebaleares.es/general/report.php?id=58&lang=es_ES [22.10.2020].
- Yunker, C.E. Studies on the Snake Mite, Ophionyssus natricis, in Nature. Science **1956**, *124*, 979–980.
- Zahradnik, P. Fauna Europaea: Gastrallus. In Fauna Europaea: Coleoptera, Anobiidae. Audisio, P. (edit.); Fauna Europaea version 2017.06. https://fauna-eu.org [29/01/2021].
- Zaragoza, J.A. Catálogo de los Pseudoescorpiones de la Península Ibérica e Islas Baleares (Arachnida: Pseudoscorpiones). Revista Ibérica de Aracnología **2006**, *13*, 3–91.

**Plant References**

- Balaguer, P.; Gómez-Pujol, L.; Fornós, J.J. 1240 Acantilados con vegetación de las costas mediterráneas con *Limonium spp*. endémicos. In: Fornós, J.J. (coord.), *Bases ecológicas preliminares para la conservación de los tipos de hábitat de interés comunitario en España*. Madrid: Ministerio de Medio Ambiente y Medio Rural y Marino, **2009**, 1–66.
- Bibiloni, G.; Alomar, G.; Rita, J. XII. Flora vascular dels illost I addicions a la flora de Cabrera Gran. In, *Història Natural de l’Arxipèlag de Cabrera*; Alcover, J.A.; Ballesteros, E.; Fornós, J.J. (Eds.) CSIC–Editorial Moll, Monografies de la Societat d’Història Natural de les Balears **1993**, *2*, 179–206.
- Cano, M.J.; Gallego, M.T.; Garilleti, R.; Juaristi, R.; Lara, F., Martínez, J.; Mazimpaka, V.; Rosselló, J.A.; Sánchez-Moya, M.C.; Urdíroz, A. Aportaciones al conocimiento de la flora briológica española. Nótula XIII: Hepáticas y musgos de Mallorca (Islas Baleares). *Boletín de la Sociedad Española de Briología* **2001**, 18/19, 103–110.
- Casas, C.; Cros, R.M.; Muñoz, J. *Triquetrella arapilensis* y especies afines: Su morfología y distribución geográfica. *The Bryologist* **1993**, 96(1), 122–131.
- Cirujano, S. Tamaricaceae. In. *Flora Ibérica*, Castroviejo, S. (coord.), **2005**, III, 437–445.
- Cros, R.M. Algunos briofitos interesantes para la flora balear. *Acta Botánica Malacitana* **1982**, 7: 141–150.
- Cros, R.M.; Brugués, M.; Sérgio, C.; Infante, M.; Heras, P. *Ephemerum recurvifolium*. In: Brugués, M., Cros, R.M. and Sérgio, C. (coord.). Cartografia de Briòfits. Península Ibèrica i Illes Balears, Available at http://briofits.iec.cat [11-03-2021].
- Herbari Virtual del Mediterrani Occidental. Available at http://herbarivirtual.uib.es/ [30-08-2021].
- Infante, M.; Sérgio, C.; Heras, P.; Cros, R.M.; Brugués, M. *Ephemerum sessile*. In Cartografia de Briòfits. Península Ibèrica i Illes Balears. Brugués, M.; Cros, R.M.; Sérgio, C. (coord.); **2019**, Available at http://briofits.iec.cat [11-03-2021].
- Puche, F.; Rosselló, J.A. XI. Flora Briològica i Pteridològica. In, *Història Natural de l’Arxipèlag de Cabrera*; Alcover, J.A., Ballesteros, E. and Fornós, J.J. (Eds.) CSIC–Editorial Moll, Monografies de la Societat d’Història Natural de les Balears **1993**, *2*, 175–178.
- Pujadas, A.J. *Daucus*. In: Castroviejo, S. (coord.) *Flora Ibérica*, **2003**, *X*, 97–125.
- Rita, J.; Bibiloni, G. XIII. La vegetació (Memòria del mapa de les comunitats vegetals). In, *Història Natural de l’Arxipèlag de Cabrera*; Alcover, J.A.; Ballesteros, E.; Fornós, J.J. (Eds.) CSIC–Editorial Moll, Monografies de la Societat d’Història Natural de les Balears **1993**, *2*, 207–255.
- Röser, M. *Stipellula*, a new genus, and new combination in feather grasses (Poaceae tribe Stipeae). *Schlechtendalia* **2012**, *24*, 91–93.
- Sáez, L.; Brugués, M.; Casas, C.; Cros, R.M; Balaguer, P. Briófitos nuevos o interesantes para las Islas Baleares. *Boletín de la Sociedad Española de Briología* **2006**, *28*, 11–23.
- SITIBSA-GOIB. Projecte BioAtles. Last updated 17/08/2021. Conselleria de Medi Ambient i Territori, Govern de les Illes Balears. 2018, Available at: http://bioatles.caib.es/serproesfront/VisorServlet [31/08/2020].
- Stevens, P.F. Angiosperm Phylogeny Website. Version 14. Last updated 06/02/2021. 2017, Available at: http://www.mobot.org/MOBOT/research/APweb/ [12/03/2021].
- Talavera, S. Cullen. In Flora Ibérica; Castroviejo, S. (coord.); 2000, VII: 357–360.
- Talavera, S. Cymodocea. Flora Ibérica, Talavera, S., Gallego, M.J.; Romero, C.; Herrero, A. (eds.) 2010, XVII: 104–107.
- Traveset, A.; Rita, J. Els illots de l’Arxipèlag de Cabrera: Refugis de biodiversitat. In Arxipèlag de Cabrera: Historia Natural. Mallorca; Grau, A.M; Fornós, J.J.; Mateu, G.; Oliver, P.A.; Terrasa, B. (eds.); Monografies de la Societat d’Historia Natural de les Balears **2020**, *30*, 513–533.
- Vives, J. Vegetació briofítica. Impressions sobre la vegetació de l’illa de Cabrera. *Treballs de la Institució Catalana d’Historia Natural* **1976**, *7*, 119–121.
- World Flora Online. Beta L. 2021, Available at www.worldfloraonline.org/taxon/wfo-4000004539. [26-07-2021].
